# Development and validation of clinical-radiomics deep learning model based on MRI for endometrial cancer molecular subtypes classification

**DOI:** 10.1186/s13244-025-01966-y

**Published:** 2025-05-16

**Authors:** Wenyi Yue, Ruxue Han, Haijie Wang, Xiaoyun Liang, He Zhang, Hua Li, Qi Yang

**Affiliations:** 1 Department of Radiology, Beijing Chaoyang Hospital, Capital Medical University, Beijing, China; 2 Department of Gynecology and Obstetrics, Beijing Chaoyang Hospital, Capital Medical University, Beijing, China; 3 Institute of Research and Clinical Innovations, Neusoft Medical Systems Co., Ltd, Beijing, China; 4 Department of Radiology, Obstetrics and Gynecology Hospital, Fudan University, Shanghai, China

**Keywords:** Endometrial cancer, Molecular subtypes, Radiomics, Deep learning, Magnetic resonance imaging

## Abstract

**Objectives:**

This study aimed to develop and validate a clinical-radiomics deep learning (DL) model based on MRI for endometrial cancer (EC) molecular subtypes classification.

**Methods:**

This multicenter retrospective study included EC patients undergoing surgery, MRI, and molecular pathology diagnosis across three institutions from January 2020 to March 2024. Patients were divided into training, internal, and external validation cohorts. A total of 386 handcrafted radiomics features were extracted from each MR sequence, and MoCo-v2 was employed for contrastive self-supervised learning to extract 2048 DL features per patient. Feature selection integrated selected features into 12 machine learning methods. Model performance was evaluated with the AUC.

**Results:**

A total of 526 patients were included (mean age, 55.01 ± 11.07). The radiomics model and clinical model demonstrated comparable performance across the internal and external validation cohorts, with macro-average AUCs of 0.70 vs 0.69 and 0.70 vs 0.67 (*p* = 0.51), respectively. The radiomics DL model, compared to the radiomics model, improved AUCs for POLEmut (0.68 vs 0.79), NSMP (0.71 vs 0.74), and p53abn (0.76 vs 0.78) in the internal validation (*p* = 0.08). The clinical-radiomics DL Model outperformed both the clinical model and radiomics DL model (macro-average AUC = 0.79 vs 0.69 and 0.73, in the internal validation [*p* = 0.02], 0.74 vs 0.67 and 0.69 in the external validation [*p* = 0.04]).

**Conclusions:**

The clinical-radiomics DL model based on MRI effectively distinguished EC molecular subtypes and demonstrated strong potential, with robust validation across multiple centers. Future research should explore larger datasets to further uncover DL’s potential.

**Critical relevance statement:**

Our clinical-radiomics DL model based on MRI has the potential to distinguish EC molecular subtypes. This insight aids in guiding clinicians in tailoring individualized treatments for EC patients.

**Key Points:**

- Accurate classification of EC molecular subtypes is crucial for prognostic risk assessment.
- The clinical-radiomics DL model outperformed both the clinical model and the radiomics DL model.
- The MRI features exhibited better diagnostic performance for POLEmut and p53abn.

**Graphical Abstract:**

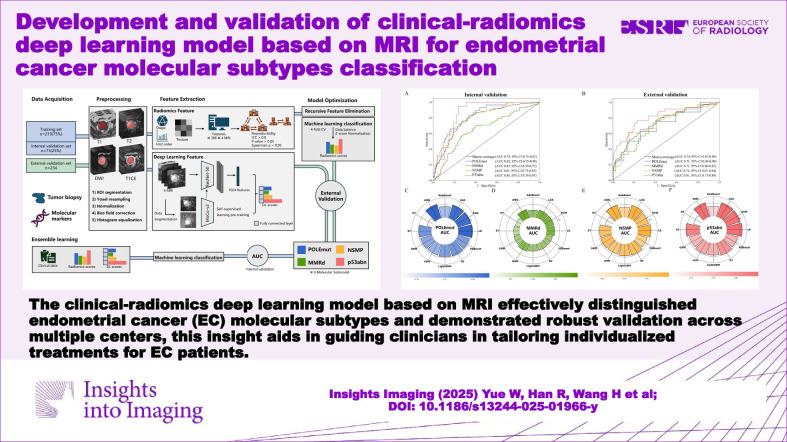

## Introduction

Endometrial cancer (EC) is one of the most common cancers, with its incidence rising globally [1]. The shift from the 2008 to the 2023 International Federation of Gynecology and Obstetrics (FIGO) guidelines incorporated molecular subtypes into EC staging. According to FIGO 2023 staging guidelines, molecular typing for all EC patients is recommended whenever feasible. This stratifies prognostic risk groups, aiding in prognostication, treatment guidance, precise surgical assessment, and the selection of adjuvant treatment strategies [2]. The Cancer Genome Atlas (TCGA) classified EC into four molecular subtypes according to genomic structure: polymerase epsilon-ultramutated (POLEmut), mismatch repair deficiency (MMRd), no specific molecular profile (NSMP), and p53-abnormal (p53abn) [3]. Each molecular subtype exhibits unique tumor biology and prognosis [4]. Patients diagnosed with POLEmut have a remarkably favorable prognosis, while those with p53abn have a less favorable prognosis. Clinical outcomes for MMRd and NSMP patients are intermediate, varying with disease stage [5]. Nevertheless, due to constraints in economic resources, testing technology, and the need for invasive procedures, there is no assurance that each EC patient will receive the molecular typing diagnosis.

Magnetic resonance imaging (MRI) provides excellent soft tissue contrast, pathological features, and molecular information [6]. However, conventional MRI lacks the specificity and sensitivity to distinguish EC molecular subtypes. Radiomics represents an innovative approach for evaluating the association between imaging features and molecular features characteristics, predicting specific endpoints such as diagnosis, treatment response, survival, as well as genomic and proteomic changes [7, 8]. In addition, deep learning (DL) autonomously extracts quantitative representations from medical images. DL radiomics has emerged as a cutting-edge quantitative tool, showcasing its capability to determine tumor subtypes [9, 10]. Previous studies have reported radiomics-based prediction of gene mutation status in ovarian cancer, breast cancer, and gliomas [11–13]. However, research on the non-invasive prediction of molecular subtypes or gene expression signature of EC using radiomics combined DL models remains limited, with validation across multiple centers yet to be accomplished [14, 15].

Our objective was to explore a clinical-radiomics DL model based on preoperative MRI to classify the EC molecular subtypes using molecular pathological diagnosis as a reference standard and validate across three institutions.

## Materials and methods

### Study participants

**Fig. 1:**
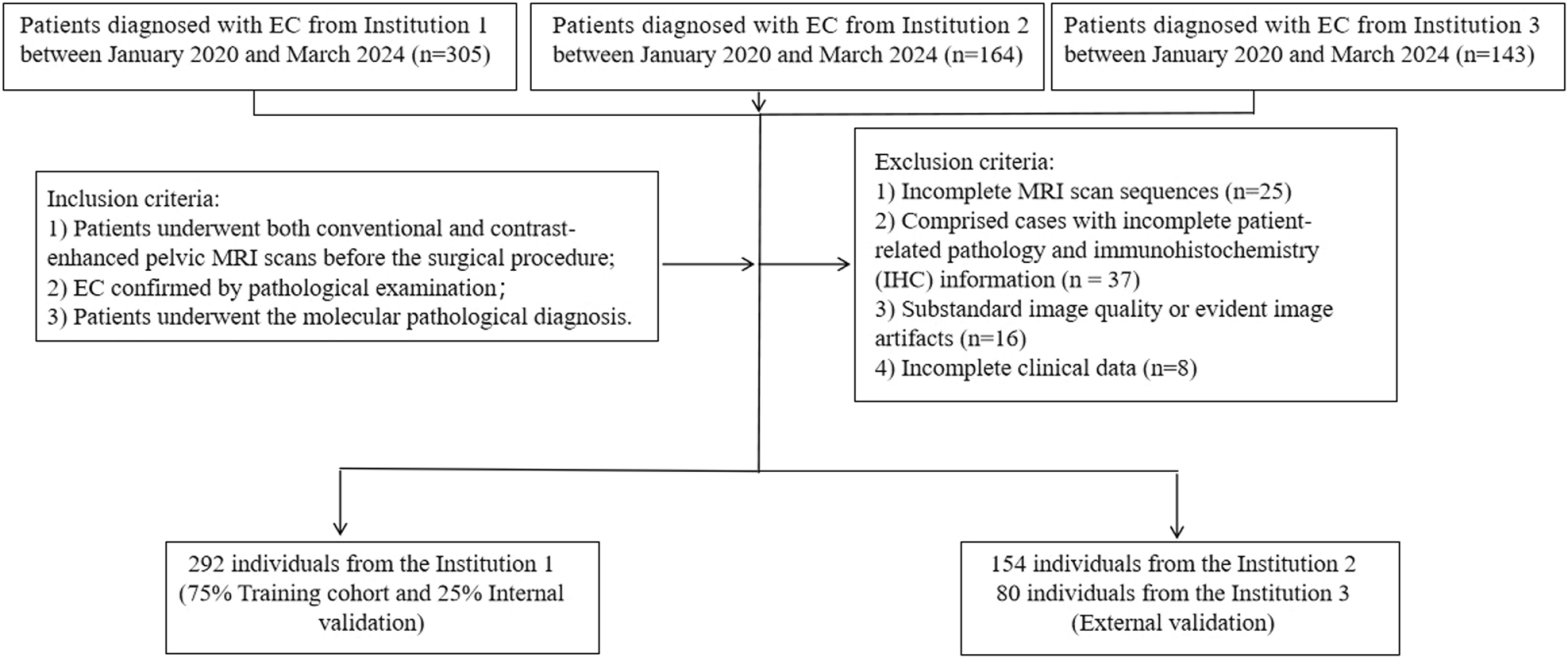
Flowchart of patient selection at the three recruiting institutions (training and validation sets). EC, endometrial cancer

This retrospective multicenter study received approval from the Institutional Review Boards of all three centers, and the need to obtain informed consent was waived due to its retrospective nature. Between January 2020 and March 2024, we reviewed patients’ records across three medical centers and included 612 patients with histopathologically confirmed EC consecutively. After applying exclusion criteria, 526 patients with EC were eligible for the study and divided into training set, internal validation, and external validation sets. Figure 1 illustrates the detailed inclusion/exclusion criteria and enrollment process.

The inclusion criteria were: (a) patients underwent both conventional and contrast-enhanced pelvic MRI scans before surgery; (b) EC was confirmed by pathological examination; and (c) Patients underwent the molecular pathological diagnosis.

The exclusion criteria were: (a) patients with incomplete pathology and immunohistochemistry information; (b) incomplete MRI scans; (c) substandard image quality affecting lesion observation and data measurement; and (d) incomplete clinical data.

The baseline clinical data including age, carbohydrate antigen (CA) 125, body mass index (BMI), menopausal status, diabetes, histopathological type, endometrioid carcinoma (EEC) grade/aggressive histological type, depth of myometrial invasion (MI) status, lymphovascular space invasion (LVSI) status, lymph node metastasis (LNM) status, and FIGO stage were gathered. The staging follows the FIGO 2023 definition without molecular subtypes [16].

### Molecular pathological preparation, reading, and diagnosis

Total genomic DNA was extracted from formalin-fixed, paraffin-embedded (FFPE) tumor tissue using the FFPE DNA kit (Amoy Diagnostics). The BPTMplus Panel (Amoy Diagnostic; Burnstone Medical) generated sequencing libraries including POLE, TP53, MLH1, MSH2, MSH6, PMS2, CTNNB1, BRCA1/2, and 55 microsatellite loci for detecting single nucleotide variants (SNVs), insertions and deletions (InDels), as well as microsatellite instability (MSI) status. Subsequent to library quality assessment, the prepared libraries were subjected to sequencing using a 2 × 150 bp paired-end approach on the Illumina NextSeq 500 platform. The generated sequence data was analyzed using the AmoyDx NGS Data Analysis System (Amoy Diagnostics; Burnstone Medical) to identify SNVs and InDels with a detection sensitivity at a variant allele frequency of ≥ 5%, as well as MSI status. The MSI status of a tumor sample was determined based on the proportion of unstable microsatellite loci, without the requirement for a paired normal control. A tumor sample was classified as MSI-positive if more than 15% of the microsatellite loci exhibited instability.

### MRI protocol and image preprocessing

All patients from the three medical centers underwent preoperative pelvic MRI scanning with both a 1.5-T MR scanner (MAGNETOM Avanto, Siemens Healthcare) and a 3.0-T MR scanner (MAGNETOM Prisma, Siemens Healthcare).

Axial T1WI, axial/sagittal/coronal fat-saturation T2-weighted imaging (T2WI), diffusion-weighted imaging (DWI) (*b*-value = 800 s/mm^2^ or 1000 s/mm^2^), and dynamic contrast enhanced T1-weighted imaging (DCE-T1WI) were employed for analysis. For DCE-T1WI, the gadolinium-based contrast agent (Magnevist, Bayer Schering) was administered at a dose of 0.2 mL/kg body weight and a rate of 2–3 mL s^−1^. The detailed MRI acquisition protocols are summarized in Table S1.

Tumor segmentation was manually conducted utilizing ITK-SNAP software (version 3.8.0, www.itksnap.org). The regions of interest (ROIs) were delineated along the tumor borders on every image slice across all sequences by Radiologist 1, who possesses 10 years of experience in gynecological imaging. Starting from axial fat-saturation T2WI, tumor volume and body contour across sequences were verified, followed by slice-by-slice segmentation across all sequences. A month later, a random selection of 50 patients was made, and the ROIs were re-delineated by the two radiologists who hold 10 years of experience in gynecological imaging, including Radiologist 1. Intraclass correlation coefficients (ICCs) quantified consistency in tumor delineation. Image preprocessing included bias field correction, pixel min–max normalization, and histogram equalization.

### Radiomics and DL feature extraction

Our study utilized the standardized reporting CLEAR checklist to achieve a standardized scientific framework, and we have also incorporated the METRICS tool in the Supplementary material to ensure that our methods and design are transparent [17]. Radiomics features were extracted using the IBSI-compliant Pyradiomics 3.0.1 Python package. A total of 386 handcrafted features were extracted per ROI from each MR sequence. Features robust to variations in contour delineation and image preprocessing parameters (ICC > 0.80), showing no significant differences across medical centers, and with low inter-feature correlation (Spearman ρ < 0.95) were retained for subsequent analysis.

**Fig. 2:**
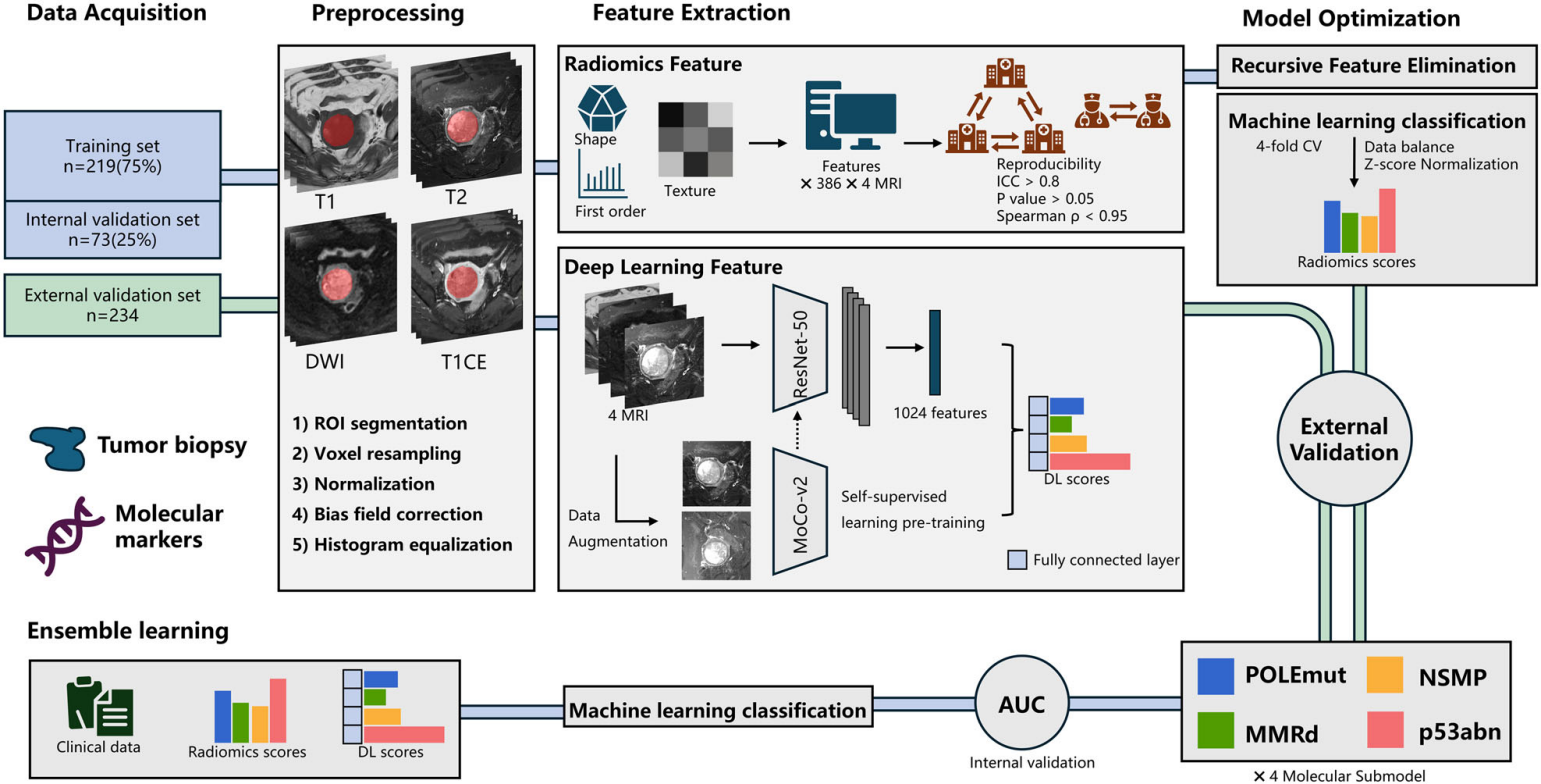
Overview of the flowchart. POLEmut, polymerase epsilon-ultramutated; MMRd, mismatch repair deficiency; NSMP, no specific molecular profile; p53abn, p53-abnormal; AUC, area under the ROC curve

DL features were extracted by employing the im4MEC method with a pretrained ResNet-50 network architecture proposed by Sarah et al [5]. The ROI containing the largest transverse area of the entire tumor was cropped, and the four sequence images were concatenated along the channel dimension. Input images were resized to 224 × 224 pixels using linear interpolation. The feature extractor model was then trained with contrastive self-supervised learning, momentum contrast for unsupervised visual representation learning-version 2 (MoCo-v2) [18], resulting in 2048 DL features extracted from 4 MR images per patient. MoCo-V2 is a method for learning visual representations without supervision, which builds on the concept of momentum contrast to improve the quality of learned features. The feature extraction process and the overview of the flowchart are illustrated in Fig. 2.

### Features selection and radiomics DL model building

Feature selection and scoring were conducted based on the training cohort, and their validity was confirmed through internal and external validation cohorts. Initially, students’ *t*-test or Mann–Whitney *U*-test identified features with an adjusted *p* value < 0.05, normalized using *z*-score. Recursive feature elimination (RFE) further refined the most predictive features. Finally, to select the best algorithms, the selected features were incorporated into 12 machine learning (ML) methods, including adaptive boosting (AdaBoost), artificial neural network (ANN), decision tree (DT), extra tree (ET), gradient boosting machine (GBM), K-nearest neighbor (KNN), linear discriminant analysis (LDA), light gradient boosting machine (LightGBM), logistic regression (LR), random forest (RF), support vector machine (SVM), and extreme gradient boosting (XGBoost), to construct radiomics DL models. Radiomics and DL scores were generated independently. To address class imbalance, random oversampling was employed during training. For the classification of four molecular subtypes, a one-vs-all strategy was applied. To identify the best model hyperparameters, a combination of the grid search technique and 4-fold cross-validation was applied. Additional details are provided in the Appendix S1.

### Development of the clinical-radiomics DL model

The clinical model was developed using univariable and multivariable LR analyses to identify significant clinical factors in predicting molecular subtypes. Clinical factors with a *p* value < 0.05 were selected for the clinical model. These clinical factors were then combined with the scores from the radiomics model and DL Model to create the clinical-radiomics DL model. The final model integrated clinical, radiomics, and DL features to improve prediction accuracy.

### Statistical analysis

Data analyses were conducted using Python version 3.11 (https://www.python.org). The area under the receiver operating characteristic curve (AUC), accuracy, sensitivity, and specificity were employed for the evaluation of each model. The SHapley Additive explanation (SHAP) method was employed for global and local model explanations. Global explanations provided consistent attribution values for each feature within the model, elucidating associations between input features and molecular subtypes. Local explanations offered specific predictions for individual cases based on input data [19]. Statistical comparisons of model performance employed the DeLong test, presenting results as mean ± SD. Comparison of clinical and pathological characteristics between EC patients from three institutions was achieved using the χ2 test, *t*-test, analysis of variance, and Fisher's exact test by SPSS 26.0 software package. *p* < 0.05 was considered indicative of a statistically significant difference.

## Results

### Participant characteristics

A total of 292, 154, and 80 patients were eligible at institutions 1, 2, and 3, respectively. 75% of the participants from Institution 1 were selected as the training set (*n* = 219), and the remaining 25% from Institution 1 were used as the internal validation set (*n* = 73). The combined total from institution 2 and institution 3 was used as the external validation set (*n* = 234). The clinical and pathological characteristics are summarized in Table 1 and Table 2. The mean age of patients in three institutions was 53.36 ± 11.61, 58.49 ± 9.63, and 54.31 ± 10.19 years, respectively. There were significant differences in age, BMI, menopausal status, history of diabetes, EEC grade, and FIGO stage between the three institutions. Institution 1 included 29 patients with POLEmut, 106 patients with MMRd, 93 patients with NSMP, and 64 patients with p53abn. Institution 2 had 8 patients with POLEmut, 30 patients with MMRd, 92 patients with NSMP, and 24 patients with p53abn. Institution 3 included 17 patients with POLEmut, 12 patients with MMRd, 41 patients with NSMP, and 10 patients with p53abn. There were significant differences in age, BMI, menopausal status, histopathological type, EEC grade, depth of MI, LVSI, LNM, and FIGO stage between different molecular subtypes.

**Table 1:** Clinical and pathological characteristics of patients with EC in the recruited patients from the training, validation, internal validation, and external validation cohorts

Clinicopathological parameters	Institution 1 (*n* = 292)	Institution 2 (*n* = 154)	Institution 3 (*n* = 80)	*p* value
Age (median, range)	53.36 ± 11.61	58.49 ± 9.63	54.31 ± 10.19	< 0.001
BMI	24.60 ± 4.57	26.45 ± 4.39	25.18 ± 4.03	< 0.001
CA125	51.83 ± 130.84	67.57 ± 273.45	24.72 ± 28.17	0.22
Menopausal status (yes)	162 (55.5%)	113 (73.4%)	50 (62.5%)	0.001
Diabetes, (%)	54 (18.5%)	49 (31.8%)	11 (13.8%)	0.001
Pathology				0.69
EEC	228 (78.1%)	128 (83.1%)	71 (88.8%)	< 0.001
Grade 1 (low grade)	98 (43.0%)	28 (21.9%)	29 (40.8%)	
Grade 2 (intermediate grade)	45 (19.7%)	78 (60.9%)	36 (50.7%)	
Grade 3 (high grade)	85 (37.3%)	22 (17.2%)	6 (8.5%)	
Carcinosarcoma	9 (3.1%)	2 (1.3%)	2 (2.5%)	
Serous carcinoma	17 (5.8%)	8 (5.2%)	1 (1.3%)	
Clear cell carcinoma	10 (3.4%)	4 (2.6%)	1 (1.3%)	
Mixed carcinoma	20 (6.8%)	9 (5.8%)	3 (3.8%)	
Poorly differentiated carcinoma	4 (1.4%)	2 (1.3%)	2 (2.5%)	
Other	4 (1.4%)	1 (0.6%)	0 (0.0%)	
MI				0.21
None	14 (4.8%)	11 (7.1%)	8 (10.0%)	
< 50%	171 (58.6%)	85 (55.2%)	51 (63.7%)	
≥ 50%	107 (36.6%)	58 (37.7%)	21 (26.3%)	
LVSI				< 0.001
None	161 (55.1%)	108 (70.1%)	60 (75.0%)	
Focal	96 (32.9%)	41 (26.6%)	20 (25.0%)	
Substantial	35 (12.0%)	5 (3.2%)	0 (0.0%)	
Lymph node status				0.14
Negative	244 (83.6%)	136 (88.3%)	73 (91.3%)	
Positive	48 (16.4%)	18 (11.7%)	7 (8.7%)	
Overall FIGO stage				< 0.001
Stage I	97 (33.2%)	74 (48.1%)	53 (66.3%)	
Stage II	106 (36.3%)	44 (28.6%)	14 (17.5%)	
Stage III	78 (26.7%)	27 (17.5%)	10 (12.5%)	
Stage IV	11 (3.8%)	9 (5.8%)	3 (3.7%)

**Table 2:** Clinicopathological characteristics of patients with four molecular subtypes of EC

Clinicopathological parameters	POLEmut (*n* = 54)	MMRd (*n* = 148)	NSMP (*n* = 226)	p53abn (*n* = 98)	*p* value
Age (median, range)	51.85 ± 7.97	54.75 ± 9.57	54.46 ± 11.99	58.39 ± 11.75	0.003
BMI	24.17 ± 4.94	24.20 ± 3.71	26.06 ± 4.72	25.46 ± 4.49	< 0.001
CA125	26.89 ± 35.39	39.58 ± 111.09	49.81 ± 176.04	91.34 ± 278.43	0.08
Menopausal status (yes)	23 (42.6%)	87 (58.8%)	141 (62.4%)	74 (75.5%)	0.001
Diabetes, *n* (%)	8 (14.8%)	28 (18.9%)	52 (23.0%)	26 (26.5%)	0.29
Pathology					< 0.001
EEC	47 (87.0%)	123 (83.1%)	207 (91.6%)	50 (51.0%)	< 0.001
Grade 1 (low grade)	16 (34.0%)	37 (30.1%)	93 (44.9%)	9 (18.0%)	
Grade 2 (intermediate grade)	11 (23.4%)	40 (32.5%)	94 (45.4%)	14 (28.0%)	
Grade 3 (high grade)	20 (42.6%)	46 (37.4%)	20 (9.7%)	27 (54.0%)	
Carcinosarcoma	1 (1.9%)	3 (2.0%)	2 (0.9%)	7 (7.1%)	
Serous carcinoma	0 (0.0%)	0 (0.0%)	1 (0.4%)	25 (25.5%)	
Clear cell carcinoma	1 (1.9%)	3 (2.0%)	4 (1.8%)	7 (7.1%)	
Mixed carcinoma	5 (9.3%)	10 (6.8%)	10 (4.4%)	7 (7.1%)	
Poorly differentiated carcinoma	0 (0.0%)	6 (4.1%)	0 (0.0%)	2 (2.0%)	
Other	0 (0.0%)	3 (2.0%)	2 (0.9%)	0 (0.0%)	
MI					0.008
None	7 (13.0%)	5 (3.4%)	15 (6.6%)	6 (6.1%)	
< 50%	28 (51.9%)	100 (67.6%)	134 (59.3%)	45 (45.9%)	
≥ 50%	19 (35.2%)	43 (29.1%)	77 (34.1%)	47 (48.0%)	
LVSI					0.02
None	32 (59.3%)	77 (52.0%)	161 (71.2%)	59 (60.2%)	
Focal	18 (33.3%)	55 (37.2%)	51 (22.6%)	33 (33.7%)	
Substantial	4 (7.4%)	16 (10.8%)	14 (6.2%)	6 (6.1%)	
Lymph node status					0.01
Negative	47 (87.0%)	127 (85.8%)	204 (90.3%)	75 (76.5%)	
Positive	7 (13.0%)	21 (14.2%)	22 (9.7%)	23 (23.5%)	
Overall FIGO stage					< 0.001
Stage I	24 (44.4%)	49 (33.1%)	133 (58.8%)	18 (18.4%)	
Stage II	18 (33.3%)	60 (40.5%)	45 (15.9%)	41 (41.8%)	
Stage III	11 (20.4%)	36 (24.3%)	39 (17.3%)	29 (29.6%)	
Stage IV	1 (1.9%)	3 (2.1%)	9 (4.0%)	10 (10.2%)	

### Clinical model and radiomics model

**Fig. 3:**
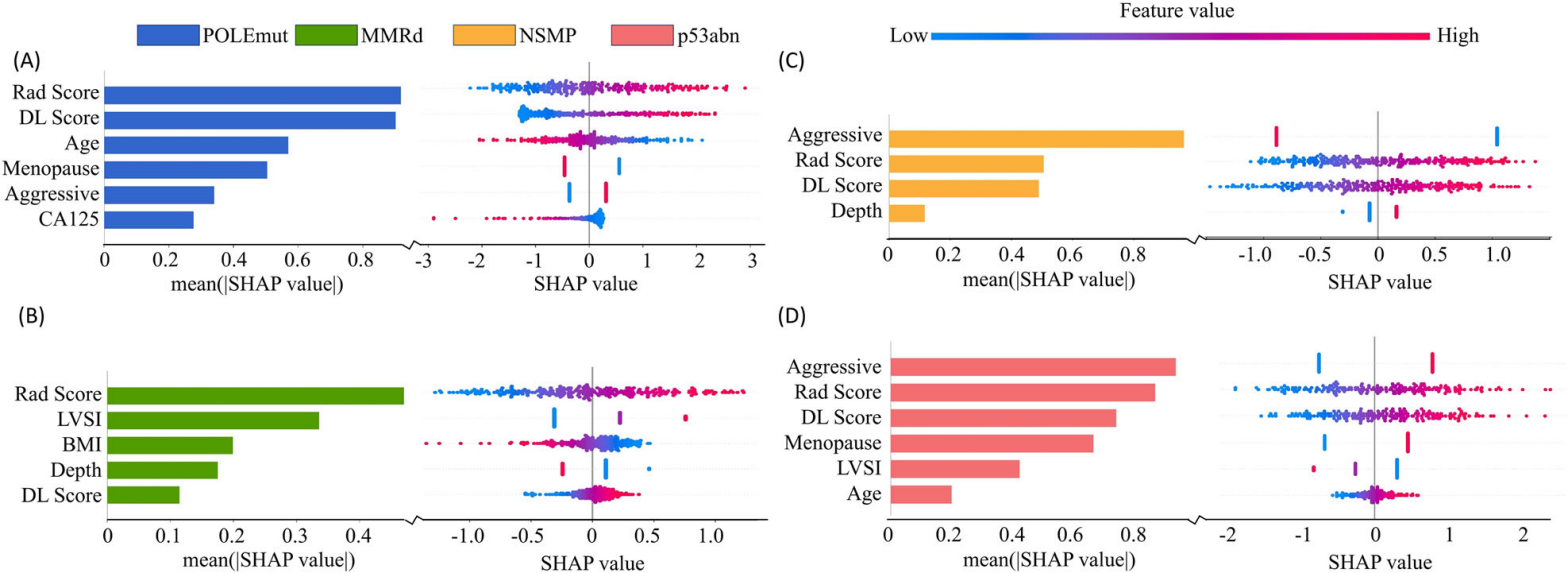
Global model explanation by the SHAP method. **A**–**D** Left figures: SHAP summary bar plot. **A**–**D** Right figures: SHAP summary dot plot. The probability of EC molecular subtypes classification increases with the SHAP value of a feature. Each patient in the model is represented by a dot corresponding to their SHAP value, with one dot per feature. The colors of these dots reflect the feature values: red indicates higher values, while blue indicates lower values. These dots are arranged vertically to indicate density. Mean (|SHAP value|) represents the average impact magnitude on the model output

In the clinical model, clinical and pathological characteristics contributed differently to the prediction of molecular subtypes (Fig. 3). Significant clinical features associated with POLEmut included age, menopausal status, aggressive histological type, and CA125 levels. For MMRd, significant contributors were LVSI status, BMI, and depth of MI. Only aggressive histological type and depth of MI were significant clinicopathological predictors for the NSMP subtype. P53abn were associated with the broadest range of predictive clinicopathological features, including aggressive histological type, menopausal status, LVSI status, age, LNM status, CA125 levels, FIGO stage, and BMI.

In Table 3, the class-wise AUCs for the clinical model were reported for each of the four molecular subtypes, with corresponding receiver operating characteristic (ROC) curves shown in Fig. S1. The clinical model achieved a macro-average AUC of 0.69 (95% CI: 0.64–0.73) in the internal validation cohort and 0.67 (95% CI: 0.60–0.73) in the external validation cohort.

**Table 3:** The class-wise AUCs of clinical model, radiomics model, DL model, radiomics DL model, and clinical-radiomics DL model in different molecular subtyping of EC

Molecular subtypes	Clinical model (AUC, 95% CI)	Radiomics model (AUC, 95% CI)	DL model (AUC, 95% CI)	Radiomics DL model (AUC, 95% CI)	Clinical-radiomics DL model (AUC, 95% CI)
POLEmut					
Internal validation	0.68 (0.57–0.78)	0.68 (0.57–0.78)	0.76 (0.67–0.84)	0.79 (0.70–0.86)	0.82 (0.75–0.90)
External validation	0.60 (0.42–0.79)	0.73 (0.64–0.82)	0.64 (0.46–0.85)	0.70 (0.47–0.90)	0.71 (0.49–0.90)
MMRd					
Internal validation	0.56 (0.50–0.63)	0.66 (0.59–0.72)	0.61 (0.55–0.68)	0.62 (0.56–0.69)	0.65 (0.59–0.72)
External validation	0.63 (0.52–0.72)	0.67 (0.57–0.78)	0.61 (0.46–0.76)	0.67 (0.60–0.79)	0.71 (0.60–0.82)
NSMP					
Internal validation	0.74 (0.68–0.80)	0.71 (0.64–0.77)	0.66 (0.59–0.72)	0.74 (0.67–0.80)	0.81 (0.75–0.85)
External validation	0.68 (0.59–0.76)	0.69 (0.57–0.81)	0.65 (0.57–0.75)	0.69 (0.56–0.80)	0.73 (0.61–0.84)
p53abn					
Internal validation	0.77 (0.63–0.88)	0.76 (0.67–0.86)	0.68 (0.61–0.76)	0.78 (0.72–0.84)	0.86 (0.78–0.93)
External validation	0.76 (0.66–0.85)	0.72 (0.63–0.81)	0.57 (0.44–0.69)	0.69 (0.59–0.79)	0.81 (0.71–0.89)
Average classification					
Internal validation	0.69 (0.64–0.73)	0.70 (0.66–0.75)	0.68 (0.64–0.72)	0.73 (0.70–0.76)	0.79 (0.75–0.82)
External validation	0.67 (0.60–0.73)	0.70 (0.64–0.75)	0.62 (0.54–0.69)	0.69 (0.62–0.76)	0.74 (0.67–0.80)

A total of 30 features were selected from the four multimodal MRI datasets, including 3 selected features from T2WI, 5 features from T1WI, 8 features from DWI, and 14 features from DCE-T1WI. The radiomics results for each single MRI sequence were shown in Table S2. Among the 12 ML models tested, the LR algorithm was selected as the final model due to its superior performance. Table S3 lists the radiomic features selected by the RFE algorithm, which were included in the four radiomics sub-models, along with their corresponding MRI sequences and feature weights in the best-performing LR model.

The radiomics model achieved a macro-average AUC of 0.70 (95% CI: 0.66–0.75) in the internal validation cohort and 0.70 (95% CI: 0.64–0.75) in the external validation cohort, as shown in Fig. S1. The specific results for the 12 ML methods, including AdaBoost, ANN, DT, ET, GBM, KNN, LDA, LightGBM, LR, RF, SVM, and XGBoost, were shown in Table S4.

The class-wise AUCs of the radiomics model across the four sub-models were presented in Table 3, with the corresponding ROC curves shown in Fig. S1. The performance of the radiomics model was similar to the clinical model, with no statistically significant differences (*p* = 0.51).

### DL model and radiomics DL model

The DL model achieved a macro-average AUC of 0.68 (95% CI: 0.64–0.72) in the internal validation cohort and 0.62 (95% CI: 0.54–0.69) in the external validation cohort. The class-wise AUCs and ROC curves are shown in Table 3 and Fig. S1.

The radiomics DL model achieved a macro-average AUC of 0.73 (95% CI: 0.70–0.76) in the internal validation cohort and 0.69 (95% CI: 0.62–0.76) in the external validation cohort, as shown in Fig. S1. The class-wise AUCs for the radiomics DL model were as follows: 0.79 (95% CI: 0.70–0.86) and 0.70 (95% CI: 0.47–0.90) for POLEmut; 0.62 (95% CI: 0.56–0.69) and 0.67 (95% CI: 0.60–0.79) for MMRd; 0.74 (95% CI: 0.67–0.80) and 0.69 (95% CI: 0.56–0.80) for NSMP; 0.78 (95% CI: 0.72–0.84) and 0.69 (95% CI: 0.59–0.79) for p53abn, in both the internal and external validation cohorts (Table 3 and Fig. S1). The radiomics DL model which added the DL model compared to the radiomics model, the performance was improved for POLEmut (AUC increased from 0.68 to 0.79, *p* = 0.001), NSMP (AUC increased from 0.71 to 0.74, *p* = 0.09) and p53abn (AUC increased from 0.76 to 0.78, *p* = 0.15) in internal validation, but performed poorly in MMRd.

### Clinical-radiomics DL model

**Fig. 4:**
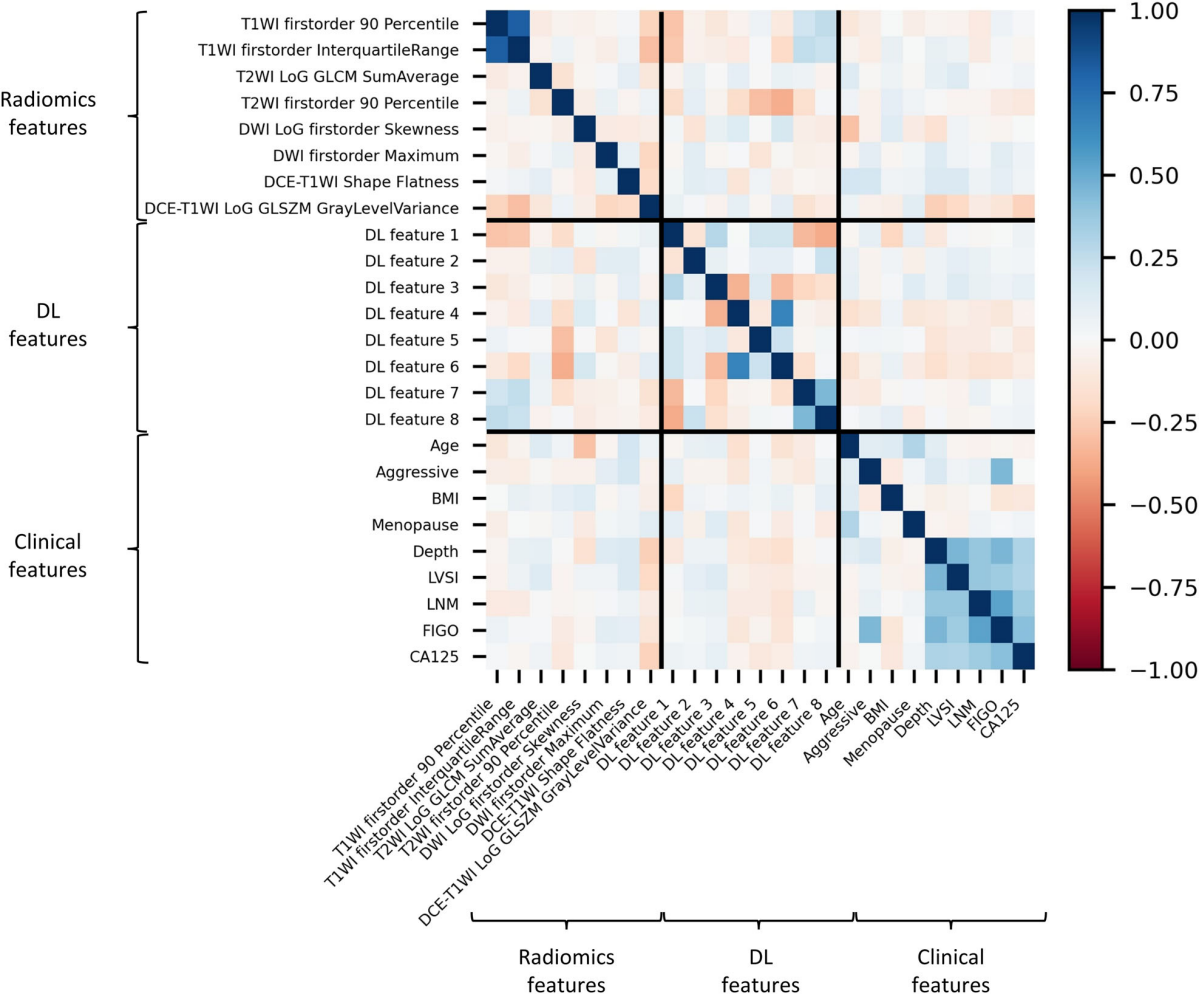
Correlation heatmap of clinical factors and top two radiomics/DL features selected with SHAP values for four molecular subtypes. DL, deep learning; BMI, body mass index; LVSI, lymphovascular space invasion; LNM, lymph node metastasis

The diagnostic performance of the best-performing clinical-radiomics DL Model for each molecular subtype in three cohorts is shown in Table 4. The class-wise AUCs of the ensemble model, which combines 12 ML models for different molecular subtypes of EC, are shown in Table S5. There was no strong correlation (Spearman rank coefficient < |0.5|) between the selected clinical, radiomics, and DL factors in the training cohort (Fig. 4).

**Table 4:** Diagnostic performance of the best-performing clinical-radiomics DL model for each molecular subtype in internal and external validation

Molecular subtypes	AUC (95% CI)	Sensitivity (%)	Specificity (%)	Accuracy (%)
POLEmut				
Internal validation	0.82 (0.75–0.90)	90	60	63
External validation	0.71 (0.49–0.90)	59	68	66
MMRd				
Internal validation	0.65 (0.59–0.72)	58	73	67
External validation	0.71 (0.60–0.82)	77	60	63
NSMP				
Internal validation	0.81 (0.75–0.85)	84	64	71
External validation	0.73 (0.61–0.84)	61	85	73
p53abn				
Internal validation	0.86 (0.78–0.93)	95	76	80
External validation	0.81 (0.71–0.89)	76	77	77

**Fig. 5:**
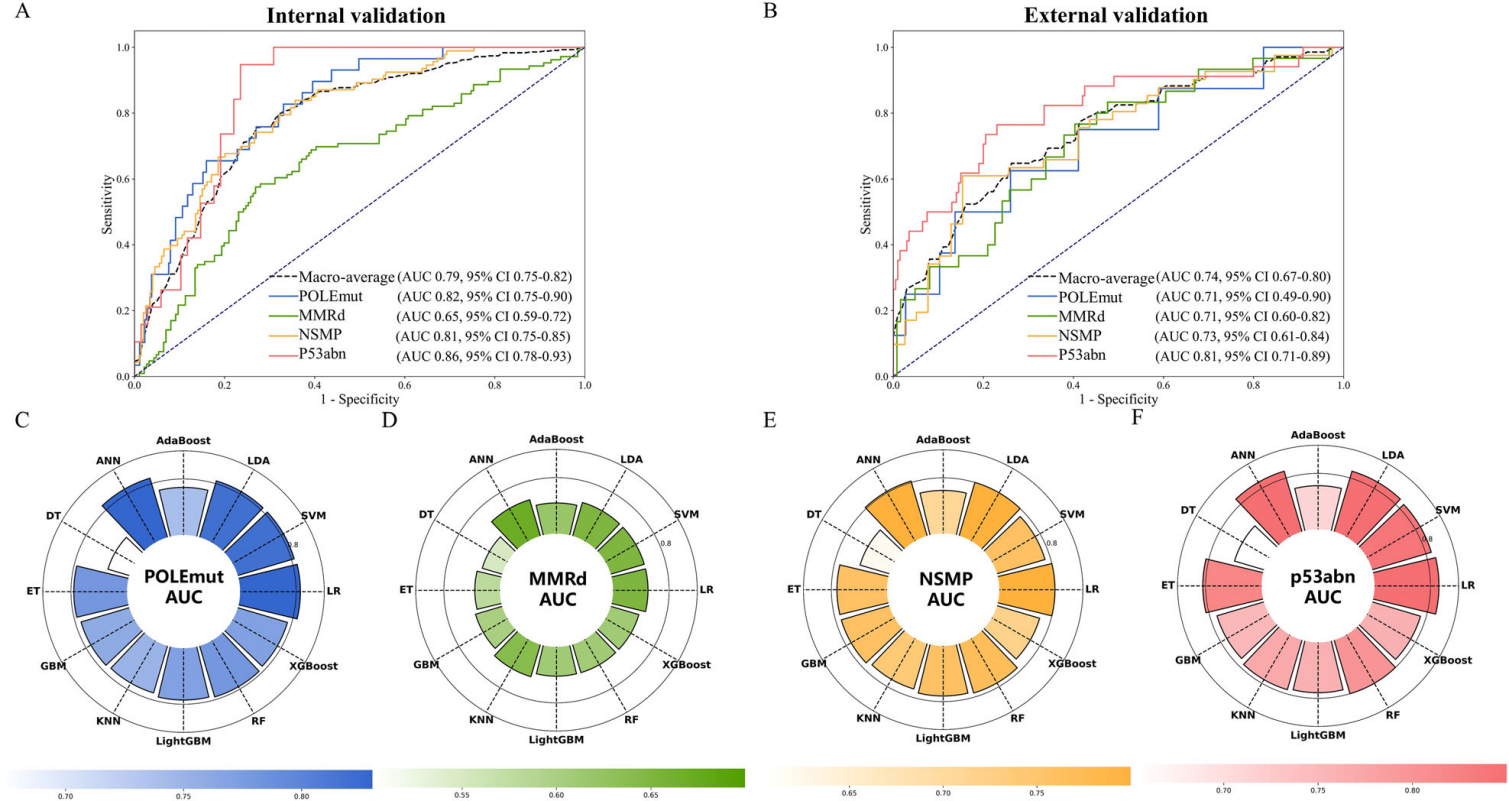
The ROC curves represent the Clinical-Radiomics DL Model (**A**, **B**) in the internal validation and external validation cohorts. The radar plot showing the area under the curve (AUC) value of each machine learning algorithm in internal validation cohorts for POLEmut (**C**), MMRd (**D**), NSMP (**E**), and p53abn (**F**). AdaBoost, adaptive boosting; ANN, artificial neural network; DT, decision tree; ET, extra tree; GBM, gradient boosting machine; KNN, K-nearest neighbor; LDA, linear discriminant analysis; LightGBM, light gradient boosting machine; LR, logistic regression; RF, random forest, SVM, support vector machine; XGBoost, extreme gradient boosting

**Fig. 6:**
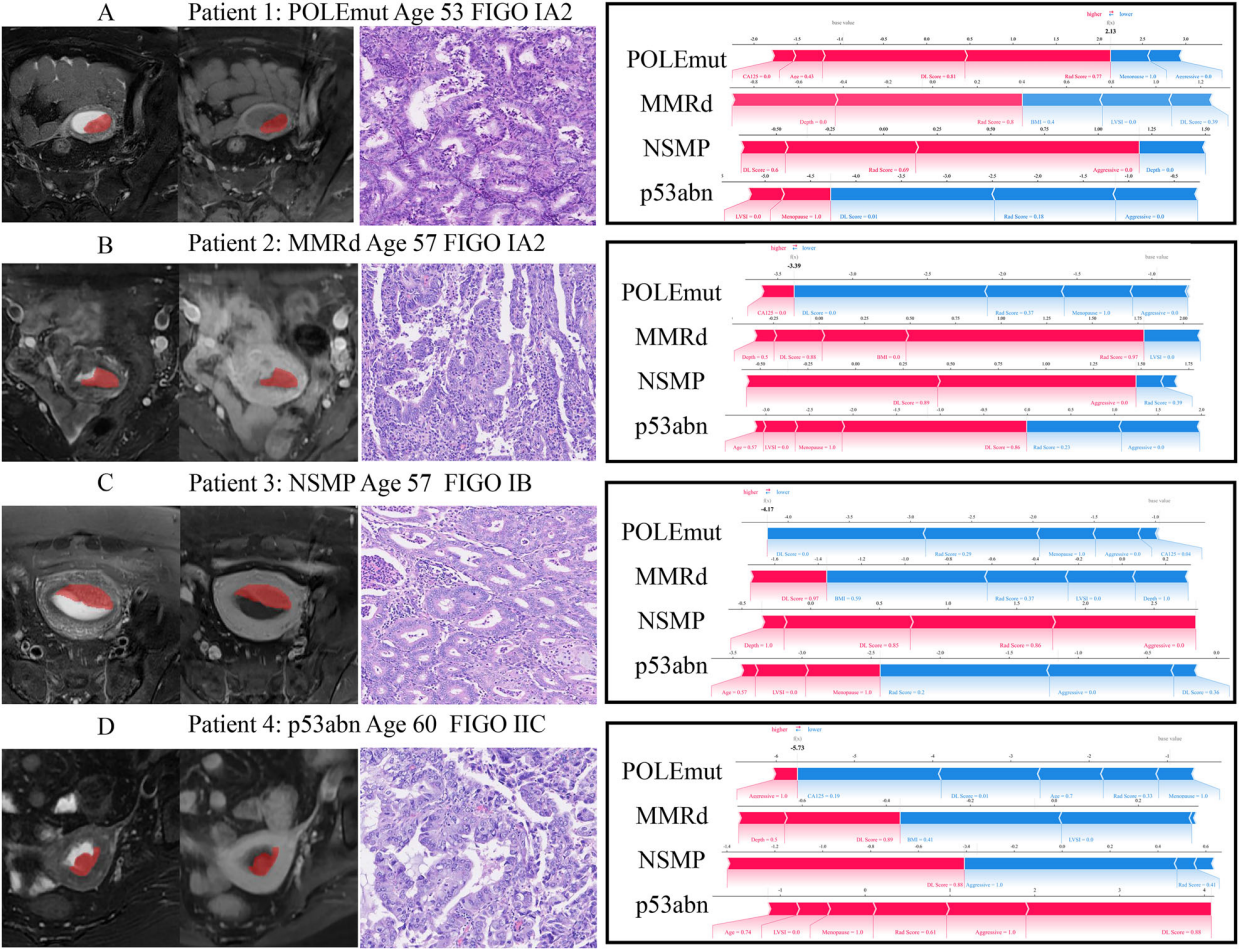
Each EC molecular subtype with corresponding MRI, histological images, and force-plot based on SHAP methods. **A** Axial view of T2WI, in a 53-year-old woman with FIGO stage IA2 EC with POLEmut. **B** Axial view of T2WI, in a 57-year-old woman with FIGO stage IA2 EC with MMRd. **C** Axial view of T2WI, in a 57-year-old woman with FIGO stage IB EC with NSMP. **D** Axial view of T2WI, in a 60-year-old woman with FIGO stage IIC EC with p53abn. The application of the final model for molecular subtypes prediction. The force plot for individual patients indicates the features that contribute to the decision of POLEmut, MMRd, NSMP, and p53abn: the blue features on the right will decrease the prediction probability, while the red feature on the left side will increase the prediction probability. POLEmut, polymerase epsilon-ultramutated; MMRd, mismatch repair deficiency; NSMP, no specific molecular profile; p53abn, p53-abnormal

The clinical-radiomics DL Model achieved the best results, with a macro-average AUC of 0.79 (95% CI: 0.75–0.82) and 0.74 (95% CI: 0.67–0.80) in the internal and external validation was shown in Fig. 5. These results are significantly better than those of the Radiomics DL Model (*p* = 0.04, 0.05). The class-wise AUCs were 0.82 (95% CI: 0.75–0.90) and 0.71 (95% CI: 0.49–0.90) for POLEmut; 0.65 (95% CI: 0.59–0.72) and 0.71 (95% CI: 0.60–0.82) for MMRd; 0.81 (95% CI: 0.75–0.85) and 0.73 (95% CI: 0.61–0.84) for NSMP; and 0.86 (95% CI: 0.78–0.93) and 0.81 (95% CI: 0.71–0.89) for p53abn in the internal and external validation. Figure 6 shows the force plot of model predictions based on SHAP methods.

## Discussion

In this study, the clinical-radiomics DL model achieved the optimal potential in classifying molecular subtypes of EC utilizing multiparametric MRI. Our preliminary result demonstrated that the clinical-radiomics DL model achieved the best performance with macro-average AUC of 0.79 and 0.74 in the internal validation and external validation. Furthermore, the class-specific AUCs of the clinical-radiomics DL model were 0.82 and 0.71 for POLEmut; 0.65 and 0.71 for MMRd; 0.81 and 0.73 for NSMP; 0.86 and 0.81 for p53abn in the internal and external validation. Notably, the identification and validation of different models highlight their potential in predicting the molecular subtypes of EC, with their robustness consistently demonstrated through external validation. Overall, our results indicated that the preoperative MRI has the potential to assist clinicians in accurately assigning patients to their respective molecular subtype classifications before surgery. In 2023, the FIGO staging system for EC was revised [16], recommending molecular subtype classification testing for all EC patients where feasible. The molecular classification of EC can potentially reduce over- and under-treatment and improve patient prognosis. Studies have shown that FIGO2023 staging combined with molecular classification contributes to the prognostic outcomes of EC patients [3, 20]. However, molecular classification validations are costly and invasive. Furthermore, the limited tissue obtained through biopsy can be insufficient, making diagnosis challenging [21]. Additionally, monitoring molecular subtypes is difficult without available tissue for mutational analysis. Therefore, identifying a non-invasive, cost-effective, and convenient method for stratifying molecular classifications in EC is important and clinically valuable.

In recent years, several studies utilizing radiomics have focused on predicting the aggressive behavior of EC. Studies using MRI radiomics have made reliable predictions of high-risk pathological types, advanced stages, deep MI and LVSI status, and the risk stratification in EC patients, but the majority of these lack an external independent validation set [1, 22–27]. Our findings align with previous results that MRI-based radiomics, alone or combined with clinical parameters, effectively predict preoperative pathologic status in EC patients. As molecular typing becomes another important factor in the prognosis of EC patients, predictive research on molecular typing has also become one of the research hotspots. There are studies on molecular classification prediction based on MRI radiomics, but most of the existing studies focus on a single subtype [15, 28]. Compared with previous studies, we added a DL model to the radiomics model to obtain a radiomics-DL model with a better performance than previously studied models, which has been validated in an external institution. In addition, we developed the clinical-radiomics DL model based on the patients’ clinical pathological information and achieved the best performance for discrimination of molecular subtypes of EC by utilizing multiparametric MRI. This also shows that when the patient’s clinical pathological information is available, the model can make better predictions for different molecular typing. For gynecologists and pathologists, it is difficult to accurately infer a patient’s molecular subtypes based on clinical and pathological information alone. However, after adding clinical pathological information to the radiomics-DL model, the clinical-radiomics DL model achieved the best performance. To our knowledge, our study has been the first multicenter study aimed at conducting molecular subtypes classification based on multimodal MRI. Building upon previous research, our study has added multicenter validation and combined multiple ML methods in a model validation, validating the feasibility of MRI-based ensemble learning for molecular subtypes of EC.

For the radiomics model, the DWI glrlm LongRunEmphasis (LRE) had the highest weight in distinguishing the p53abn subtype. LRE emphasizes the presence of long runs, indicating that in DWI, p53abn tumors exhibit more intense water molecule activity. This was consistent with the p53abn subtype, which represents the most heterogeneous EC molecular subtype. The DL model significantly enhanced the diagnostic performance of POLEmut and p53abn in the internal validation. However, its contribution to the diagnostic performance of MMRd was lower. This could be attributed to the fact that POLEmut and p53abn patients often correspond to the better and the worse prognoses, respectively, and these characteristics were more prominently displayed in single-center MRI images. As a result, the self-supervised model may overly focus on these related features, thereby improving the internal diagnostics for POLEmut and p53abn while neglecting learning for MMRd and NSMP. The performance of the DL model on external validation sets was weaker than radiomics model, which is due likely to the fact that compared to manually designed radiomics features, the method of automatic feature extraction in DL had a higher potential but also more difficulty in achieving good robustness in medical research with relatively small sample sizes. For POLEmut and MMRd, image-based Rad Score/DL Score had the highest mean SHAP value, indicating the highest average impact on model output magnitude. These patients exhibited a more stable clinical presentation and showed more prominent features on imaging. In contrast, the higher weight of the aggressive histological type for p53mut aligns with clinical cognition of a worse prognosis in patients.

The current study has a few limitations that should be noted. First, our external validation cohorts were relatively small since the relatively few patients with EC who have undergone molecular pathological diagnosis and have received molecular subtype results. Second, our patients exhibited an imbalance in the classification numbers of molecular subtypes of EC, particularly with fewer POLEmut patients. Although we employed random oversampling in the training cohort, achieving a balanced distribution of data will enhance the model’s performance. Third, the delineation of ROIs was manually performed by radiologists, and there are still certain deviations among the three institutions, which may be alleviated by developing DL algorithms for automatic lesion segmentation, therefore minimizing ROI deviations. Fourth, we applied MRI image data scanned on either 1.5-T or 3.0-T MR scanners in our study, with a relatively small sample size. Additionally, due to the absence of ADC images for the majority of our data, we were unable to include the relevant quantitative information of ADC maps in our analysis. This diversity, along with the lack of ADC images, may lead to unfavorable heterogeneity in subsequent results and contribute to the lower robustness of our DL model. In future research, we will endeavor to collect patients with complete sequence data to address this limitation. Finally, the prognosis differs among the four molecular subtypes. Our model’s predictions regarding prognosis require further prospective validation, which is also a focus of our future research direction.

## Conclusion

Our radiomics DL model has enabled the MRI-based EC molecular subtypes' non-invasive classification. The clinical-radiomics DL model performed better when clinical pathological information was available. Subsequent exploration should be conducted with prospective validation and a larger dataset to better guide clinicians in developing personalized treatment plans for patients with EC.

## Supplementary information

ELECTRONIC SUPPLEMENTARY MATERIAL

## Data Availability

The data that supports the findings of this study are available in the supplementary material of this article.

## Abbreviations

AUC: Area under the receiver operating characteristic curve
DL: Deep learning
EC: Endometrial cancer
FIGO: International Federation of Gynecology and Obstetrics
MMRd: Mismatch repair deficiency
MoCo-v2: Momentum contrast for unsupervised visual representation learning-version 2
NSMP: No specific molecular profile
p53abn: p53-abnormal
POLEmut: Polymerase epsilon-ultramutated
ROC: Receiver operating characteristic
