# Supplementary material for: Development and validation of clinical-radiomics deep learning model based on MRI for endometrial cancer molecular subtypes classification
Source: Insights Imaging. 2025 May 16;16:107. doi: 10.1186/s13244-025-01966-y (PMC12084453; doi:10.1186/s13244-025-01966-y)
Supplement: Supplementary file 1 — ELECTRONIC SUPPLEMENTARY MATERIAL [file 13244_2025_1966_MOESM1_ESM.pdf]

# Development and validation of Clinical-Radiomics Deep Learning Model based on MRI for Endometrial Cancer Molecular Subtypes Classification
# ELECTRONIC SUPPLEMENTARY MATERIAL

**Table S1: The detailed MRI acquisition protocols.**

**Table S1a. Details of parameters for 1.5 Tesla MRI (SIEMENS) imaging protocols.**

| Sequence | Repetition Time/TR (msec) | Echo Time/TE (msec) | Field of view/FOV (mm) | Thicknes s (mm) | Gap (mm) | Flip angle (degrees) |
|---|---|---|---|---|---|---|
| **T2WI** | 4000 | 83 | 350 | 4 | 1.2 | 144 |
| **T1WI** | 550 | 10 | 350 | 4 | 1.2 | 150 |
| **DWI** | 2800 | 81 | 300 | 5 | 1.5 | 11 |
| **DCE-T1WI** | 4.89 | 2.38 | 380 | 3 | 0.9 | 10 |

**Table S1b. Details of parameters for 3.0 Tesla MRI (SIEMENS) imaging protocols.**

| Sequence | Repetition Time/TR (msec) | Echo Time/TE (msec) | Field of view/FOV (mm) | Thickness (mm) | Gap (mm) | Flip angle (degrees) |
|---|---|---|---|---|---|---|
| **T2WI** | 3700 | 100 | 100 | 7 | 8.4 | 150 |
| **T1WI** | 550 | 10 | 100 | 7 | 8.4 | 150 |
| **DWI** | 4800 | 91 | 70 | 6 | 7.8 | 90 |
| **DCE-T1WI** | 500 | 11 | 100 | 7 | 8.4 | 140 |

## Table S2: The radiomics results of the single MRI sequence.

| MRI sequence (AUC, 95% CI) | POLEmut | | MMRd | | NSMP | | p53abn | |
|---|---|---|---|---|---|---|---|---|
| **Radiomics Model** | Internal Validation | External Validation | Internal Validation | External Validation | Internal Validation | External Validation | Internal Validation | External Validation |
| **T1WI** | 0.60(0.49-0.71) | 0.65(0.45-0.84) | 0.57(0.51-0.64) | 0.54(0.36-0.71) | 0.56(0.49-0.62) | 0.60(0.51-0.69) | 0.61(0.52-0.69) | 0.63(0.51-0.75) |
| **T2WI** | 0.58(0.47-0.71) | 0.54(0.37-0.72) | 0.60(0.53-0.67) | 0.62(0.40-0.82) | 0.64(0.60-0.73) | 0.58(0.45-0.71) | 0.64(0.57-0.72) | 0.62(0.50-0.73) |
| **DWI** | 0.63(0.53-0.73) | 0.62(0.45-0.78) | 0.55(0.48-0.61) | 0.63(0.52-0.75) | 0.57(0.50-0.64) | 0.60(0.51-0.69) | 0.71(0.60-0.82) | 0.70(0.58-0.81) |
| **DCE-T1WI** | 0.67(0.55-0.78) | 0.64(0.48-0.78) | 0.60(0.53-0.67) | 0.62(0.50-0.73) | 0.63(0.55-0.70) | 0.62(0.53-0.71) | 0.75(0.63-0.87) | 0.68(0.60-0.77) |

**Table S3: The selected radiomics features and their corresponding coefficients in four LR sub-models.**

| Molecular Subtypes | Feature | Coefficient |
|---|---|---|
| POLEmut | | |
| | DWI firstorder Maximum | 11.05 |
| | DCE T1WI LoG-sigma-1-0-mm glszm GrayLevelNonUniformityNormalized | 1.97 |
| | DCE T1WI shape Flatness | 1.57 |
| | T1WI LoG-sigma-3-0-mm firstorder Median | 0.40 |
| | DCE T1WI LoG-sigma-5-0-mm glcm JointAverage | -1.76 |
| | DCE T1WI glcm InverseVariance | -1.77 |
| | DWI LoG-sigma-1-0-mm firstorder Skewness | -2.05 |
| MMRd | | |
| | T1WI firstorder 90Percentile | 2.36 |
| | T2WI LoG-sigma-5-0-mm glrlm LongRunLowGrayLevelEmphasis | 2.17 |
| | T1WI LoG-sigma-5-0-mm glcm ClusterShade | 1.63 |
| | DCE T1WI LoG-sigma-5-0-mm glcm ClusterProminence | 1.59 |
| | DWI LoG-sigma-1-0-mm gldm DependenceVariance | 1.31 |
| | DCE T1WI firstorder InterquartileRange | 1.29 |
| | DCE T1WI shape Sphericity | 1.04 |
| | DCE T1WI LoG-sigma-3-0-mm firstorder RootMeanSquared | 1.00 |
| | DCE T1WI LoG-sigma-1-0-mm firstorder Mean | 0.82 |
| | T2WI LoG-sigma-5-0-mm glcm SumAverage | -0.88 |
| | T1WI firstorder InterquartileRange | -2.88 |
| NSMP | | |
| | T2WI LoG-sigma-5-0-mm glcm SumAverage | 2.83 |
| | T1WI LoG-sigma-3-0-mm glcm Imc1 | 2.12 |

| | | |
|---|---|---|
| | T2WI LoG-sigma-5-0-mm firstorder Minimum | -2.04 |
| | DWI LoG-sigma-1-0-mm gldm DependenceVariance | -2.15 |
| | DCE T1WI shape Flatness | -2.37 |
| p53abn | | |
| | DWI original ngtdm Busyness | -8.52 |
| | DWI LoG-sigma-1-0-mm glrlm LongRunEmphasis | 8.63 |
| | DWI LoG-sigma-1-0-mm glszm SizeZoneNonUniformity | 3.56 |
| | DCE T1WI LoG-sigma-3-0-mm firstorder 90Percentile | 1.82 |
| | DCE T1WI LoG-sigma-3-0-mm glszm LargeAreaEmphasis | 1.84 |
| | DWI LoG-sigma-1-0-mm glszm LargeAreaHighGrayLevelEmphasis | -11.74 |
| | DWI LoG-sigma-5-0-mm glcm ClusterShade | 2.34 |
| | DCE T1WI LoG-sigma-5-0-mm glcm ClusterTendency | -1.46 |
| | DCE T1WI firstorder Variance | -3.74 |
| | DCE T1WI LoG-sigma-5-0-mm firstorder Minimum | -1.70 |

**Table S4: The class-wise AUCs of the radiomics model combining twelve machine learning models in different molecular subtyping of EC.**

| Molecular subtypes (AUC, 95% CI) | POLEmut | | MMRd | | NSMP | | p53abn | |
|---|---|---|---|---|---|---|---|---|
| **Radiomics Model** | Internal Validation | External Validation | Internal Validation | External Validation | Internal Validation | External Validation | Internal Validation | External Validation |
| **LR** | 0.68 (0.57-0.78) | 0.73 (0.64-0.82) | 0.66 (0.59-0.72) | 0.67 (0.57-0.78) | 0.71 (0.64-0.77) | 0.69 (0.57-0.81) | 0.76 (0.67-0.86) | 0.72 (0.63-0.81) |
| **SVM** | 0.68 (0.58-0.78) | 0.70 (0.58-0.83) | 0.64 (0.57-0.71) | 0.62 (0.50-0.73) | 0.67 (0.60-0.74) | 0.67 (0.55-0.78) | 0.71 (0.64-0.78) | 0.62 (0.49-0.73) |
| **LDA** | 0.67 (0.56-0.78) | 0.70 (0.56-0.84) | 0.64 (0.58-0.71) | 0.67 (0.57-0.78) | 0.68 (0.61-0.75) | 0.61 (0.52-0.71) | 0.65 (0.58-0.73) | 0.64 (0.53-0.74) |
| **AdaBoost** | 0.68 (0.59-0.78) | 0.60 (0.34-0.85) | 0.56 (0.49-0.62) | 0.57 (0.45-0.70) | 0.71 (0.65-0.77) | 0.63 (0.53-0.71) | 0.58 (0.49-0.67) | 0.58 (0.44-0.71) |
| **ANN** | 0.67 (0.56-0.78) | 0.69 (0.54-0.84) | 0.64 (0.57-0.70) | 0.68 (0.49-0.84) | 0.68 (0.60-0.74) | 0.69 (0.56-0.80) | 0.65 (0.57-0.73) | 0.66 (0.55-0.76) |
| **DT** | 0.51 (0.45-0.57) | 0.54 (0.46-0.69) | 0.56 (0.50-0.61) | 0.55 (0.40-0.72) | 0.57 (0.51-0.62) | 0.56 (0.46-0.65) | 0.55 (0.50-0.61) | 0.51 (0.42-0.61) |
| **ET** | 0.60 (0.47-0.73) | 0.57 (0.33-0.82) | 0.55 (0.48-0.62) | 0.57 (0.46-0.70) | 0.65 (0.59-0.72) | 0.62 (0.54-0.72) | 0.62 (0.55-0.70) | 0.58 (0.45-0.72) |
| **GBM** | 0.63 (0.52-0.73) | 0.57 (0.42-0.73) | 0.58 (0.51-0.65) | 0.60 (0.47-0.73) | 0.67 (0.61-0.74) | 0.64 (0.51-0.76) | 0.57 (0.49-0.65) | 0.59 (0.48-0.71) |
| **KNN** | 0.64 (0.53-0.76) | 0.59 (0.40-0.80) | 0.56 (0.50-0.63) | 0.56 (0.46-0.69) | 0.63 (0.56-0.69) | 0.61 (0.52-0.70) | 0.62 (0.55-0.70) | 0.53 (0.37-0.70) |
| **LightGBM** | 0.57 (0.45-0.70) | 0.61 (0.45-0.76) | 0.56 (0.49-0.63) | 0.60 (0.49-0.72) | 0.64 (0.57-0.71) | 0.61 (0.53-0.70) | 0.61 (0.52-0.70) | 0.63 (0.43-0.81) |
| **RF** | 0.59 (0.48-0.70) | 0.58 (0.36-0.78) | 0.58 (0.51-0.65) | 0.64 (0.46-0.83) | 0.65 (0.58-0.72) | 0.62 (0.53-0.71) | 0.65 (0.58-0.72) | 0.59 (0.39-0.79) |
| **XGBoost** | 0.56 (0.42-0.70) | 0.51 (0.35-0.66) | 0.57 (0.50-0.65) | 0.57 (0.48-0.68) | 0.66 (0.59-0.72) | 0.64 (0.56-0.73) | 0.62 (0.54-0.70) | 0.62 (0.51-0.73) |

**Table S5. The class-wise AUCs of the ensemble model combining twelve machine learning models in different molecular subtypes of EC.**

| **Molecular subtypes (AUC, 95% CI)** | **POLEmut** | | **MMR-d** | | **NSMP** | | **p53abn** | | **Average Classification** | |
|---|---|---|---|---|---|---|---|---|---|---|
| **Clinical-Radiomics-DL Combined Model** | Internal Validation | External Validation | Internal Validation | External Validation | Internal Validation | External Validation | Internal Validation | External Validation | Internal Validation | External Validation |
| **LR** | 0.82 (0.75-0.90) | 0.71 (0.49-0.90) | 0.65 (0.59-0.72) | 0.71 (0.60-0.82) | 0.81 (0.75-0.85) | 0.73 (0.61-0.84) | 0.86 (0.78-0.93) | 0.81 (0.71-0.89) | 0.78 | 0.74 |
| **SVM** | 0.82 (0.74-0.89) | 0.73 (0.57-0.89) | 0.65 (0.58-0.72) | 0.67 (0.49-0.83) | 0.76 (0.70-0.82) | 0.72 (0.64-0.80) | 0.84 (0.78-0.89) | 0.78 (0.67-0.88) | 0.77 | 0.73 |
| **LDA** | 0.82 (0.75-0.90) | 0.71 (0.52-0.88) | 0.65 (0.58-0.72) | 0.65 (0.49-0.80) | 0.80 (0.74-0.85) | 0.72 (0.63-0.80) | 0.85 (0.80-0.90) | 0.77 (0.65-0.87) | 0.78 | 0.71 |
| **AdaBoost** | 0.72 (0.62-0.82) | 0.60 (0.39-0.81) | 0.62 (0.56-0.68) | 0.65 (0.52-0.76) | 0.71 (0.64-0.77) | 0.64 (0.55-0.73) | 0.71 (0.63-0.79) | 0.68 (0.46-0.87) | 0.70 | 0.64 |
| **ANN** | 0.84 (0.78-0.91) | 0.71 (0.50-0.90) | 0.67 (0.61-0.74) | 0.72 (0.55-0.86) | 0.81 (0.75-0.86) | 0.72 (0.63-0.80) | 0.84 (0.80-0.89) | 0.78 (0.66-0.88) | 0.79 | 0.73 |
| **DT** | 0.56 (0.49-0.65) | 0.56 (0.49-0.71) | 0.55 (0.49-0.61) | 0.55 (0.46-0.65) | 0.62 (0.56-0.68) | 0.57 (0.49-0.65) | 0.62 (0.56-0.69) | 0.59 (0.44-0.74) | 0.59 | 0.57 |
| **ET** | 0.78 (0.70-0.87) | 0.71 (0.58-0.84) | 0.58 (0.51-0.65) | 0.59 (0.47-0.71) | 0.76 (0.70-0.81) | 0.70 (0.58-0.82) | 0.82 (0.76-0.87) | 0.78 (0.66-0.88) | 0.73 | 0.70 |
| **GBM** | 0.76 (0.68-0.85) | 0.69 (0.48-0.91) | 0.60 (0.54-0.67) | 0.59 (0.49-0.72) | 0.76 (0.69-0.82) | 0.65 (0.55-0.74) | 0.75 (0.68-0.81) | 0.75 (0.62-0.86) | 0.72 | 0.67 |
| **KNN** | 0.75 (0.65-0.84) | 0.60 (0.46-0.78) | 0.64 (0.58-0.71) | 0.65 (0.54-0.75) | 0.74 (0.68-0.80) | 0.69 (0.60-0.78) | 0.77 (0.71-0.83) | 0.74 (0.62-0.84) | 0.73 | 0.67 |
| **LightGBM** | 0.77 (0.68- | 0.69 (0.52- | 0.61 (0.55- | 0.60 (0.50- | 0.76 (0.70- | 0.65 (0.56- | 0.76 (0.70- | 0.75 (0.53- | 0.73 | 0.67 |

| | | | | | | | | | | |
|---|---|---|---|---|---|---|---|---|---|---|
| | 0.85) | 0.87) | 0.68) | 0.72) | 0.82) | 0.73) | 0.82) | 0.94) | | |
| **RF** | 0.78 (0.69-0.87) | 0.73 (0.56-0.89) | 0.61 (0.54-0.67) | 0.62 (0.44-0.80) | 0.77 (0.71-0.82) | 0.65 (0.53-0.78) | 0.80 (0.74-0.85) | 0.77 (0.65-0.88) | 0.74 | 0.69 |
| **XGBoost** | 0.77 (0.67-0.86) | 0.68 (0.45-0.90) | 0.61 (0.55-0.68) | 0.58 (0.48-0.69) | 0.72 (0.66-0.78) | 0.66 (0.54-0.78) | 0.76 (0.70-0.82) | 0.71 (0.47-0.91) | 0.72 | 0.66 |

## Table S6. METRICS Tool v1.0

| Items/Conditions | Definitions | Weights | Options |
|---|---|---|---|
| Study Design | | | |
| Item#1 | Adherence to radiomics and/or machine learning-specific checklists or guidelines | 0.0368 | yes |
| Item#2 | Eligibility criteria that describe a representative study population | 0.0735 | yes |
| Item#3 | High-quality reference standard with a clear definition | 0.0919 | yes |
| Imaging Data | | | |
| Item#4 | Multi-center | 0.0438 | yes |
| Item#5 | Clinical translatability of the imaging data source for radiomics analysis | 0.0292 | yes |
| Item#6 | Imaging protocol with acquisition parameters | 0.0438 | yes |
| Item#7 | The interval between imaging used and reference standard | 0.0292 | yes |
| Segmentation | | | |
| Condition#1 | Does the study include segmentation? | | yes |
| Condition#2 | Does the study include fully automated segmentation? | | no |
| Item#8 | Transparent description of segmentation methodology | 0.0337 | yes |
| Item#9 | Formal evaluation of fully automated segmentation | 0.0225 | n/a |
| Item#10 | Test set segmentation masks produced by a single reader or automated tool | 0.0112 | yes |
| Image Processing and Feature Extraction | | | |
| Condition#3 | Does the study include hand-crafted feature extraction? | | yes |
| Item#11 | Appropriate use of image preprocessing techniques with transparent description | 0.0622 | yes |
| Item#12 | Use of standardized feature extraction software | 0.0311 | yes |
| Item#13 | Transparent reporting of feature extraction parameters, otherwise providing a default configuration statement | 0.0415 | yes |
| Feature Processing | | | |
| Condition#4 | Does the study include tabular data? | | yes |
| Condition#5 | Does the study include end-to-end deep learning? | | yes |
| Item#14 | Removal of non-robust features | 0.0200 | yes |
| Item#15 | Removal of redundant features | 0.0200 | yes |
| Item#16 | Appropriateness of dimensionality compared to data size | 0.0300 | yes |
| Item#17 | Robustness assessment of end-to-end deep learning pipelines | 0.0200 | no |
| Preparation for Modeling | | | |
| Item#18 | Proper data partitioning process | 0.0599 | yes |
| Item#19 | Handling of confounding factors | 0.0300 | yes |
| Metrics and Comparison | | | |
| Item#20 | Use of appropriate performance evaluation metrics for task | 0.0352 | yes |
| Item#21 | Consideration of uncertainty | 0.0234 | yes |

| | | | |
|---|---|---|---|
| Item#22 | Calibration assessment | 0.0176 | no |
| Item#23 | Use of uni-parametric imaging or proof of its inferiority | 0.0117 | yes |
| Item#24 | Comparison with a non-radiomic approach or proof of added clinical value | 0.0293 | yes |
| Item#25 | Comparison with simple or classical statistical models | 0.0176 | yes |
| Testing | | | |
| Item#26 | Internal testing | 0.0375 | yes |
| Item#27 | External testing | 0.0749 | yes |
| Open Science | | | |
| Item#28 | Data availability | 0.0075 | no |
| Item#29 | Code availability | 0.0075 | yes |
| Item#30 | Model availability | 0.0075 | no |
| Total METRICS score: | | 94.6% | |
| Quality category: | | Excellent | |
| Publication ID: | | INSI-S-24-01679 | |

**Figure S1. The ROC curves represent the clinical models (A and E), deep learning/DL models (B and F), Radiomics models (C and G), Radiomics-DL Model (D and H) in internal validation and external validation cohort, respectively.**

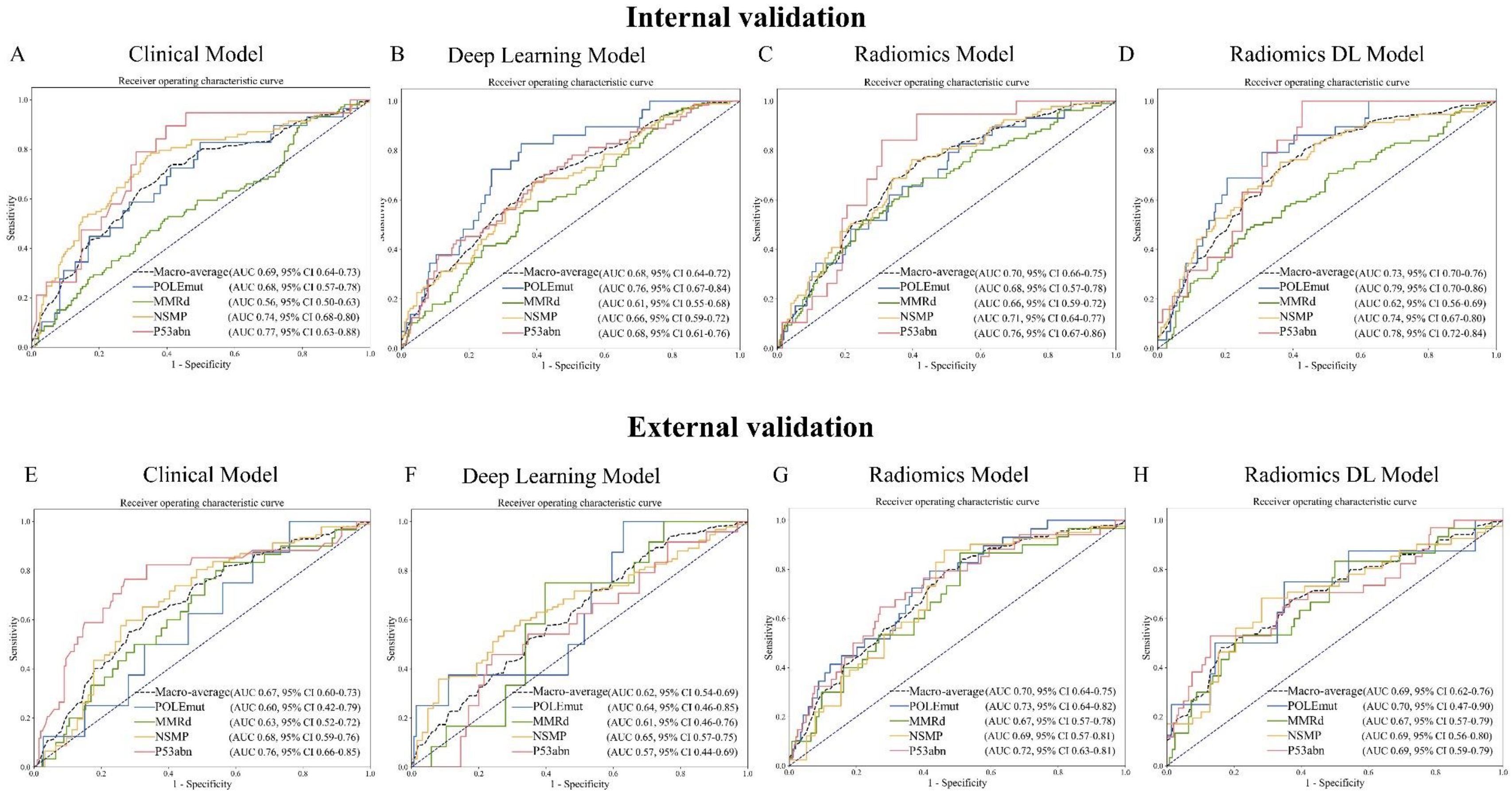

**Appendix S1**

A. Radiomics features

Before feature extraction, the voxel size of each sequence was resampled to 1 x 1 x 1 $mm^3$, and pixel intensity was min-maxnormalized to convert images into standardized inputs. These inputs had an intensity range of 0 to 1. A total of 386 quantitative imaging features including 14 shape-based features, 18 first order statistical features, 22 greyscale co-occurrence matrix (GLCM), 16 grey-level run length matrix (GLRLM), 16 grey-level size zone matrix (GLSZM), 14 grey level dependence matrix (GLDM) and 5 neighboring grey-tone difference matrix (NGTDM) features and 281 derived features based on the Laplacian-of-Gaussian (LoG) filtered images, were extracted each MR sequences using corresponding ROIs.

B. DL features

DL features were extracted by employing based on the im4MEC method proposed by Sarah et al. Code is available at https://github.com/AIRMEC/im4MEC.

C. Model construction (Machine Learning Algorithms)

To select the best performance of the model. We are using the 12 machines learning algorithms. The patients were partitioned into training and internal validation cohort randomly with a 7:3 ratio in center 1. To identify the best model hyperparameters, a combination of grid search technique and 5-fold cross-validation was applied. The top-performing models in the training cohort were then evaluated on internal and external validation 1/2 cohorts. The clinical-radiomics-DL combined model was formulated using scores from both radiomics and deep learning, the best result was processed through logistic regression using Python with SciPy (v1.4.1) and scikit-learn package (v.0.22). The code to study the full process is available at https://github.com/haijiewangmri/CRDLM_for_EC.
